# Slit in the Coronaries: A Case of Spontaneous Coronary Artery Dissection

**DOI:** 10.7759/cureus.4841

**Published:** 2019-06-05

**Authors:** Anandbir S Bath, Sourabh Aggarwal, Vishal Gupta, Jagadeesh K Kalavakunta

**Affiliations:** 1 Internal Medicine, Western Michigan University Homer Stryker M.D. School of Medicine, Kalamazoo, USA; 2 Interventional Cardiology, Ascension Borgess Hospital, Kalamazoo, USA; 3 Cardiology, Ascension Borgess Hospital, Kalamazoo, USA

**Keywords:** spontaneous coronary artery dissection (scad), acute coronary syndrome (acs), interventional cardiology

## Abstract

Spontaneous coronary artery dissection (SCAD) is a noniatrogenic epicardial coronary artery dissection unrelated to an atherosclerotic disease process. SCAD is responsible for a small percentage of acute coronary syndrome (ACS) cases. The left anterior descending (LAD) artery is the most common artery affected in SCAD, although any coronary artery can be affected. We present an interesting case of SCAD presenting as an ST-elevation myocardial infarction complicated with dissection extending to the left main and distal LAD requiring emergent coronary artery bypass grafting. Our case emphasizes the importance of considering SCAD as a cause for ACS, especially in young patients with minimal atherosclerotic risk factors. Also, a very high recurrence rate demands strict follow-up and multidisciplinary decision making in the population impacted with this rare entity.

## Introduction

Spontaneous coronary artery dissection (SCAD) is defined as a noniatrogenic epicardial coronary artery dissection that is not related to atherosclerotic plaque rupture/erosion [1]. SCAD is a rare and uncommon cause of acute coronary syndrome (ACS), which can have varied presentations such as myocardial infarction, ventricular arrhythmias, and sudden cardiac death with 2%-5% of the cases presenting as cardiogenic shock [2-6]. We present an interesting case of SCAD presenting as an ST-elevation myocardial infarction (STEMI) complicated with dissection extending to the left main and distal left anterior descending (LAD) artery requiring emergent coronary artery bypass grafting (CABG).

## Case presentation

A 48-year-old man with a known history of hypertension and no known cardiac history presented to the ED with substernal chest pain radiating to the left jaw, axilla, and arm. It was sudden in onset; he reported the pain started while he was mowing his grass and was associated with shortness of breath and diaphoresis. He had no personal history of diabetes mellitus, hyperlipidemia, smoking, or use of illicit drugs and denied any family history of premature coronary artery disease. His vital signs were significant for elevated blood pressure of 202/126 mmHg with oxygen saturation >95% on room air. He was anxious and otherwise had an unremarkable examination with normal S1 and S2 with no murmurs, rubs, or gallops and no rales or rhonchi.

**Figure 1:**
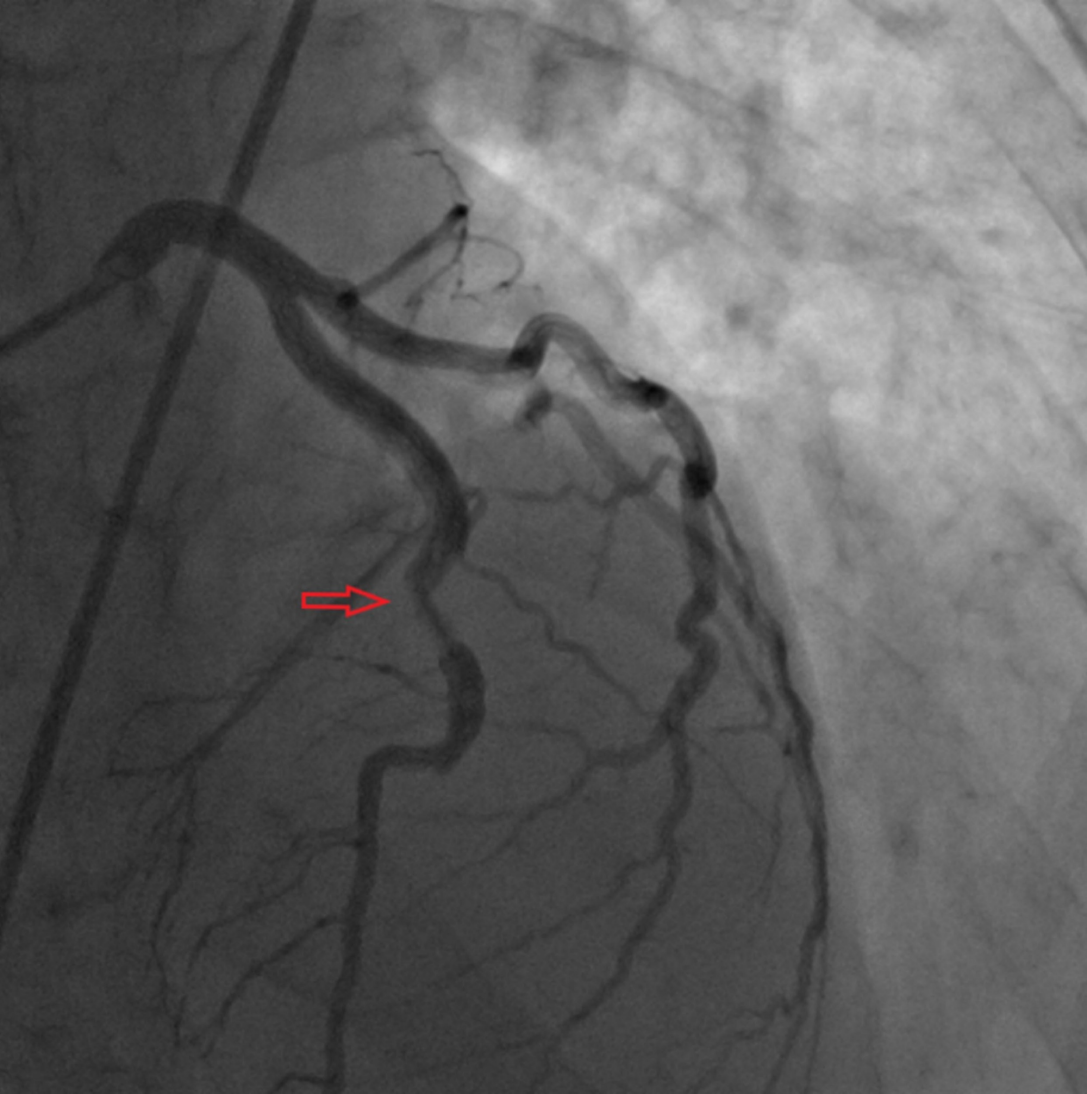
Filling defect noted in middle left anterior descending artery.

An electrocardiogram revealed ST elevations in lead I, V4-V6 suggesting anterolateral STEMI. He was loaded with aspirin 325 mg oral and 5000 units of IV heparin. He was emergently transferred to the cardiac catheterization laboratory for angiography. His coronary angiogram was done via the right femoral route and revealed right dominant coronary anatomy with no angiographic evidence of obstructive disease in the right coronary artery and left circumflex artery. His mid LAD had filling defect suspicious for plaque rupture and thrombosis (Figure 1).

After administering additional heparin to achieve activated clotting time > 250, left main was engaged with extra backup (XB) 3.5 guide catheter and short runthrough wire advanced to the distal LAD. The wire advanced easily, and we navigated to the septal and diagonal branches, confirming the intraluminal location of the wire. On follow-up angiogram, we noted dissection in his middle LAD extending to the diagonal branch, confirming the diagnosis of SCAD.

**Figure 2:**
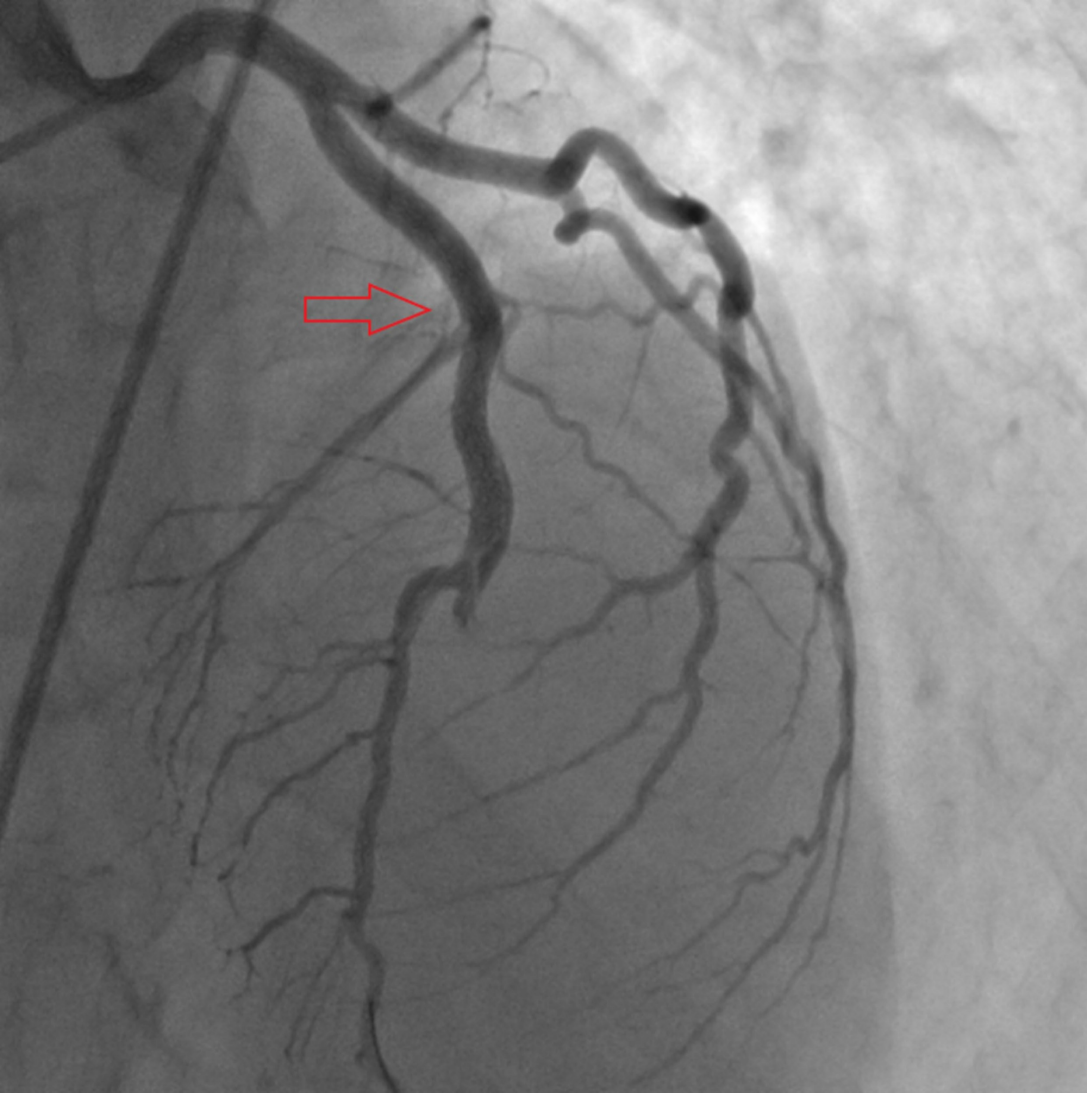
Proximal end of dissection sealed with drug-eluting stent.

We initially tried to wire the diagonal branch; however, we were unable to advance the wire to the diagonal branch. We then deployed a drug-eluting stent to seal the proximal end of dissection with a 3.5-mm x 18-mm zotarolimus-eluting stent, and we used a 2.5-mm x 23-mm zotarolimus-eluting stent to seal the distal end of the dissection (Figure 2).

**Figure 3:**
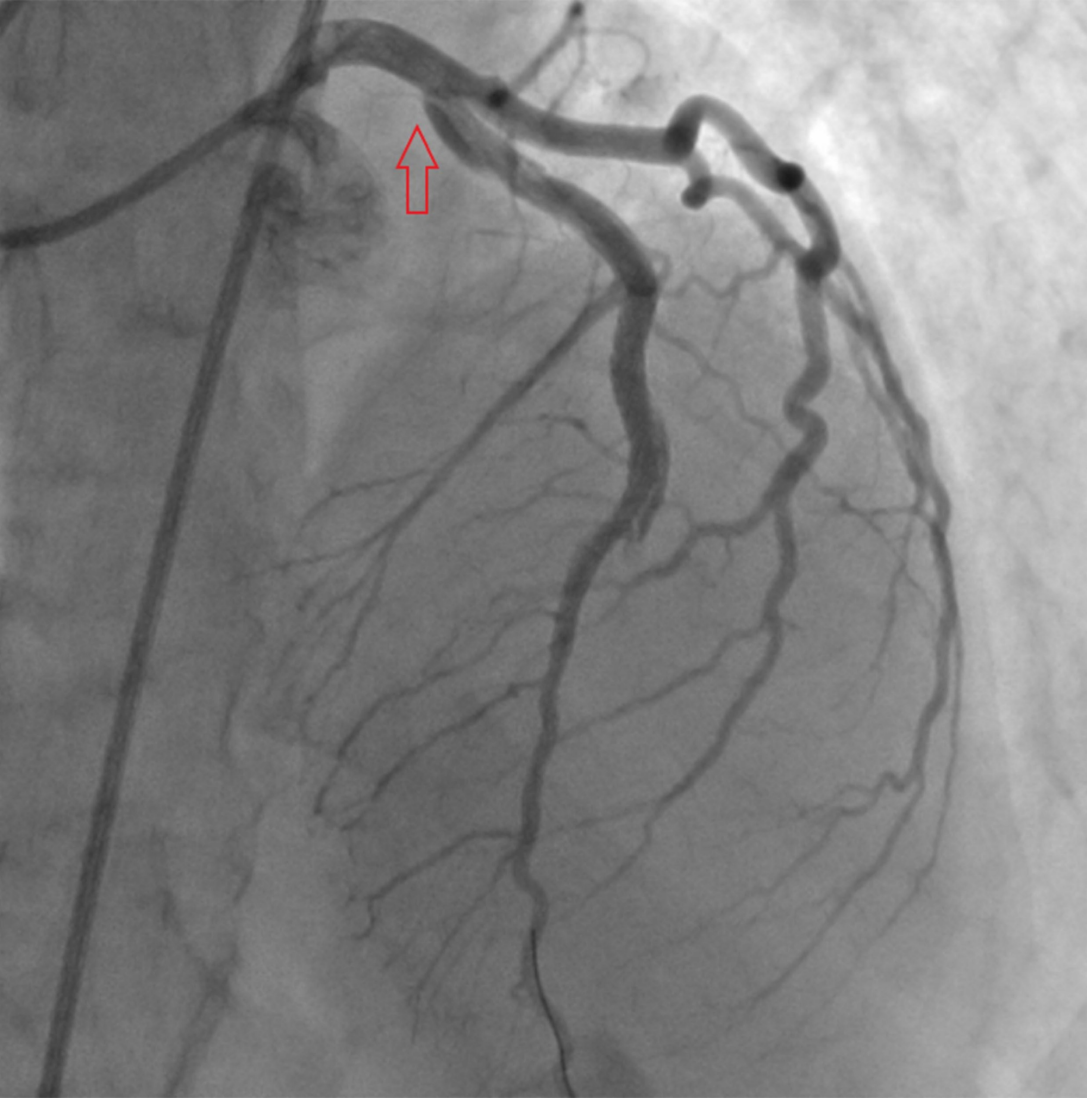
Proximal dissection extending into ostial left anterior descending artery involving the left main coronary artery.

On follow-up angiogram, we noted the proximal dissection extended to the ostial LAD involving the distal left main artery (Figure 3).

At this point, we were concerned that further intervention might lead to a further extension of the dissection proximally, jeopardizing flow to the left main/left circumflex. We consulted with the CT surgical team and discussed the case with them in detail. The patient was emergently taken to the OR for CABG and successfully underwent three-vessel CABG with left internal mammary artery to LAD, aorto-coronary saphenous vein graft to diagonal branch and aortocoronary saphenous vein graft to the obtuse marginal branch. The patient had an unremarkable postoperative course, was extubated the day after the procedure, and went home after three days. He was seen in the clinic for follow-up evaluation and was doing fine.

## Discussion

We present an interesting case of SCAD that presented with STEMI complicated by extension of the dissection proximally, necessitating emergent CABG. SCAD was initially diagnosed in 1931 during the autopsy of a 42-year-old woman. Originally thought to be prevalent in women, SCAD is usually seen in individuals with few traditional atherosclerotic risk factors. While women comprise most cases, men usually present at a younger age than their counterparts with a mean age of 49 [7-8]. Its pathogenesis involves the formation of an intramural hematoma (IMH) within the arterial wall, which further compresses the arterial lumen and leads to decreased blood flow. Two mechanisms have been described for the formation of IMH depending on the place of arterial wall separation. Both intimal tear and vasa vasorum rupture lead to pooling of blood in the intramural space, creating a false lumen. Atherosclerotic dissection of the coronaries can differ from the SCAD based on its limited nature of propagation [2]. In our case, the initial appearance was concerning for atherosclerotic plaque rupture; however, the easy extension of the dissection proximally and distally was more consistent with SCAD.

Common conditions which predispose to SCAD include fibromuscular dysplasia, pregnancy, multiparity, inherited arteriopathies, connective tissue disorders, exogenous hormone use, systemic inflammatory disease, and coronary artery spasm. More than 50% of these patients recall a precipitating factor such as intense exercise, intense Valsalva maneuver, retching, vomiting, intense emotional stress, labor, and recreational drug abuse. Chest pain is the presenting symptom in the majority of the cases with most presenting as ACS [2, 9-11]. Studies that exclude iatrogenic, traumatic, and atherosclerotic dissection suggest that SCAD may be a cause of up to 1%-4% of ACS cases overall, and SCAD is the most common cause of pregnancy-associated myocardial infarction (MI; 43%) and up to 35% of MIs in women aged 50 years or younger. The LAD is the most common artery affected in SCAD, although any coronary artery can be affected. In a recent case series, 26% of cases were found to present as STEMI and 3.6% with ventricular arrhythmia [12].

Missed diagnosis on angiography and lack of clinical familiarity account for the underdiagnoses of SCAD cases. Intracoronary imaging techniques like intravascular ultrasound (IVUS) and optical coherence tomography (OCT) have increased the detection rates for SCAD, but these are not needed to make the diagnosis [13-15]. However, operators must be careful as intracoronary placement of IVUS catheter and contrast injection during OCT imaging can cause an extension of the dissection. Because the clinical course of our case was consistent with SCAD, we elected not to intervene/interrogate further with intravascular imaging. Three distinct angiographic patterns have been described to help with diagnosis [16]. The appearance of multiple lumens (true and false) on contrast dye staining constitutes type I SCAD. Type II SCAD is characterized by diffuse stenosis of varying severity. Type III SCAD differs from the other two due to focal stenosis and its similarities with atherosclerosis, often requiring intracoronary imaging for its diagnosis. However, a high index of suspicion is needed for diagnosis.

The most recent position statement from the American Heart Association recommended conservation therapy for clinically stable patients with no high-risk features on angiography. However, percutaneous intervention or CABG is recommended in patients with active/ongoing ischemia or hemodynamic instability and CABG in stable patients with involvement of the left main and/or proximal two vessel dissection [1]. Spontaneous healing occurs in most of the cases, suggesting time-dependent healing, but extensive inpatient monitoring is recommended. Interestingly, guidelines recommend the use of the femoral route for angiography as there is a higher rate of iatrogenic dissection in patients with SCAD, and more with the radial route. Hemodynamic stability can dictate further management with unstable patients requiring urgent percutaneous intervention or CABG. In a recent review article, 84% of patients were managed conservatively, 15% with percutaneous intervention, and 1% required urgent CABG [17]. Complications such as the iatrogenic extension of the dissection and true lumen occlusion should be considered before proceeding with the early invasive strategy [5-6,18-19]. Dual antiplatelet therapy for one year is recommended after the successful percutaneous intervention [20].

Our patient presented with an ST-elevation in the anterolateral leads and had ongoing chest pain, which prompted an urgent revascularization course. He had type I SCAD on angiography. We attempted to stabilize the dissection once the guidewire was confirmed in the true lumen. The placement of the stent led to proximal and distal extension of the dissection requiring multiple stent placements and urgent cardiothoracic consultation for emergent CABG.

## Conclusions

The diagnosis of SCAD should be considered in all cases of ACS presenting in young patients with minimal traditional atherosclerotic risk factors. Hemodynamic and clinical stability should dictate the management strategies unless the patient presents with ischemic changes where urgent intervention is needed for revascularization. The 50% recurrence rate demands strict follow-up evaluation, and multidisciplinary decision making is necessary for favorable outcomes in patients impacted with this rare entity.
